# Targeting Bacterial Biofilms on Medical Implants: Current and Emerging Approaches

**DOI:** 10.3390/antibiotics14080802

**Published:** 2025-08-06

**Authors:** Alessandro Calogero Scalia, Ziba Najmi

**Affiliations:** Department of Health Sciences, Center for Translational Research on Autoimmune & Allergic Diseases, CAAD, University of Piemonte Orientale, Corso Trieste 15/A, 28100 Novara, Italy; alessandro.scalia@uniupo.it

**Keywords:** EPS, matrix eradication, medical device, non-antibiotic agents

## Abstract

Biofilms are structured communities of microorganisms encased in a self-produced extracellular matrix, and they represent one of the most widespread forms of microbial life on Earth. Their presence poses serious challenges in both environmental and clinical settings. In natural and industrial systems, biofilms contribute to water contamination, pipeline corrosion, and biofouling. Clinically, biofilm-associated infections are responsible for approximately 80% of all microbial infections, including endocarditis, osteomyelitis, cystic fibrosis, and chronic sinusitis. A particularly critical concern is their colonization of medical devices, where biofilms can lead to chronic infections, implant failure, and increased mortality. Implantable devices, such as orthopedic implants, cardiac pacemakers, cochlear implants, urinary catheters, and hernia meshes, are highly susceptible to microbial attachment and biofilm development. These infections are often recalcitrant to conventional antibiotics and frequently necessitate surgical revision. In the United States, over 500,000 biofilm-related implant infections occur annually, with prosthetic joint infections alone projected to incur revision surgery costs exceeding USD 500 million per year—a figure expected to rise to USD 1.62 billion by 2030. To address these challenges, surface modification of medical devices has emerged as a promising strategy to prevent bacterial adhesion and biofilm formation. This review focuses on recent advances in chemical surface functionalization using non-antibiotic agents, such as enzymes, chelating agents, quorum sensing quenching factors, biosurfactants, oxidizing compounds and nanoparticles, designed to enhance antifouling and mature biofilm eradication properties. These approaches aim not only to prevent device-associated infections but also to reduce dependence on antibiotics and mitigate the development of antimicrobial resistance.

## 1. Bacterial Biofilm

### 1.1. Biofilm Formation

**Figure 1:**
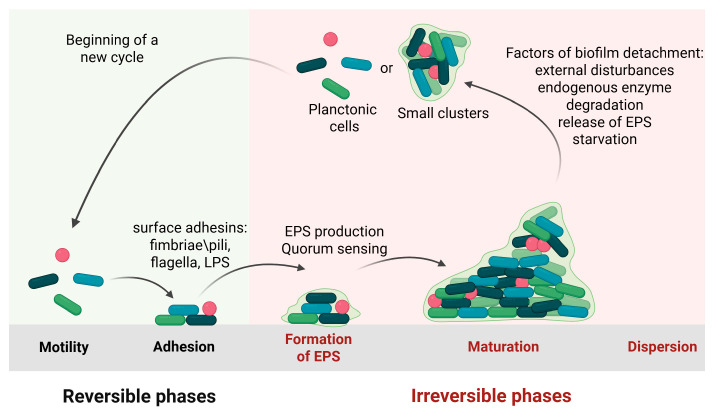
Schematic illustration of the key steps in biofilm formation on both biotic and abiotic surfaces (created in BioRender. Cochis, A. (2025) https://BioRender.com/j8kiffx, accessed on 1 August 2025).

Biofilm formation is a multi-step, complex process in which bacteria transition from a free-floating planktonic state to a sessile form. This process is influenced by various external factors, including desiccation, nutrient limitation, extreme temperature or pH, antimicrobial agents, gravitational and hydrodynamic forces, the nature of the colonized surfaces, quorum sensing, secondary messengers, and other signaling molecules [1,2]. Figure 1 illustrates the major stages of biofilm development, typically divided into four main steps:

#### 1.1.1. Initial Attachment

The first stage of biofilm formation is bacterial attachment, initiated by favorable interactions between planktonic cells and a surface. Bacterial transport to these surfaces occurs through Brownian motion, sedimentation, or convection [3]. Upon contact, adhesion is governed by a balance of attractive and repulsive interfacial force. When attractive forces dominate, attachment occurs; conversely, if repulsive forces prevail, adhesion is hindered [4]. This initial, reversible phase relies primarily on non-specific physical interactions, including electrostatic forces, hydrophobic interactions, and Lifshitz–van der Waals forces [5].

Both inert and biological surfaces can support bacterial adhesion, and virtually any material in contact with a bacterial suspension may serve as a substrate for biofilm development [6,7]. Surface properties, such as roughness and topography [7,8], hydrophobicity [8,9], surface charge [8,10], and the presence of conditioning films (layers of adsorbed nutrients and molecules) [11,12], strongly influence the efficiency and speed of bacterial attachment.

#### 1.1.2. Irreversible Adhesion and Aggregation

Irreversible bacterial attachment is governed by short-range interactions, including dipole–dipole forces, hydrogen bonds, ionic and covalent bonding, and hydrophobic interactions, all of which are facilitated by bacterial adhesins. Additionally, adhesion structures, such as flagella, pili/fimbriae, and non-fimbriae adhesins, which extend from the bacterial surface, play crucial roles in establishing initial contact with substrates [5].

Flagella, filamentous structures responsible for bacterial motility, support both swimming (in liquids) and swarming (on moist surfaces), enabling cells to move toward favorable environments and initiate surface attachment [13,14]. A study on four types of *Escherichia coli*, with and without flagella, demonstrated that flagellated strains exhibited stronger and more consistent attachment to six different types of plastics commonly found in freshwater systems [14]. Furthermore, two flagellar mutant strains of *Salmonella enteritidis*, SE-Δ*flhD* and SE-Δ*fligE*, showed reduced sedimentation rates and lower biofilm biomass compared to wild-type (WT) strains [15].

Pili/fimbriae, another class of filamentous surface appendage, contribute to intercellular interactions and early surface colonization in several bacterial species [16], including *Klebsiella pneumoniae* [17], *Streptococcus agalactiae* [18], *Clostridium difficile* [19], and *Acinetobacter baumanii* [20]. For instance, *Pseudomonas aeruginosa* utilizes pili for both surface adhesion and twitching motility [17]. In *K. pneumoniae*, both type 1 and type 3 fimbriae facilitate adhesion to abiotic surfaces and promote mature biofilm formation. In contrast, in *Erwinia amylovora*, only type 1 fimbriae are involved in this process; mutant strains lacking type 1 fimbriae exhibit significantly reduced adherence to abiotic surfaces compared to wild-type strains [17]. In addition to physical appendages, pathogens may express adhesins that bind to eukaryotic cell receptors, promoting host invasion. For example, *Yersinia pseudotuberculosis* and *Y. enterocolitica* produce invasion proteins that bind β1 integrins on M-cells, facilitating cellular entry [21].

Following irreversible adhesion, bacteria undergo multiplication and division to form microcolonies. This process is initiated by the production of extracellular polymeric substances (EPSs) [22,23,24]. EPS is a key component of the biofilm matrix, promoting cell-to-cell cohesion and surface attachment through hydrophobic and ionic bridging interactions [25,26]. In addition to structural stability, EPS plays diverse roles, including surface adherence, cellular communication, biofilm architecture formation, water retention, signaling, protection, nutrient entrapment, symbiosis, and genetic exchange. The intracellular secondary messenger cyclic di-guanosine monophosphate (c-di-GMP) further promotes the transition to irreversible adhesion by upregulating the synthesis of EPS and surface-associated structures [25]. The composition and functional roles of the extracellular matrix will be described in detail in Section 3.

#### 1.1.3. Biofilm Maturation

The third stage of biofilm formation is maturation, during which microcolonies grow and develop into more complex structures. This phase is facilitated by the secretion of signaling molecules, such as quorum sensing (QS) autoinducers and intercellular communication signals, by the attached bacterial cells, leading to the expression of biofilm-specific genes. The release of EPS plays a pivotal role in stabilizing the biofilm structure and protecting it from antimicrobial agents [26]. Following EPS production, water-filled channels form within the biofilm, functioning like a circulatory system that distributes nutrients to cells and removes waste products [27]. The maturation process typically occurs in two distinct phases: stage I involves cell-to-cell interactions and the synthesis of autoinducer signaling molecules, such as N-acylated homoserine lactone (AHL); stage II is characterized by an increase in the size and thickness of the microcolonies, reaching up to approximately 100 µm, considered a defining feature of established microcolonies [28]. During maturation, bacterial motility is restricted, and the expression of genes involved in surface structure formation is downregulated. Consequently, the gene expression profile of sessile cells within the biofilm differs significantly from that of planktonic cells. For instance, DNA microarray analyses have revealed altered expression of over 70 genes in *P. aeruginosa* after five days of biofilm growth [29]. Additionally, QS facilitates intra- and interspecies communication through the production and detection of autoinducers (AIs), small signaling molecules that allow bacteria to sense population density and coordinate gene expression accordingly [30]. The mechanisms and types of QS signals will be discussed in detail in Section 4.3.

#### 1.1.4. Dispersal/Detachment

Biofilm detachment, also known as dispersal, represents the terminal stage of biofilm development. It is considered a survival strategy, allowing bacterial cells to exit the biofilm and initiate a new cycle of biofilm formation elsewhere. Dispersal is a complex, regulated process influenced by environmental cues, signal transduction pathways, and effector molecules [31].

**Figure 2:**
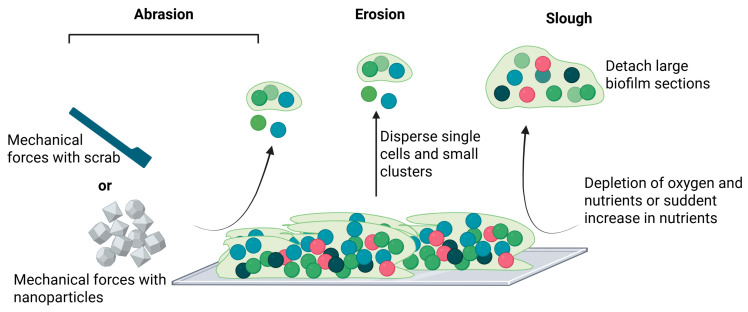
Dispersal biofilm: abrasion, erosion, and sloughing (created in BioRender. Cochis, A. (2025) https://BioRender.com/xyuiufv, accessed on 1 August 2025).

Although dispersal mechanisms vary among bacterial species, the overall process can generally be divided into three key stages: detachment of cells from the microcolonies, movement of cells to a new surface, and reattachment to initiate colonization of the new substrate [31,32]. Detachment can occur via both active and passive mechanisms: (i) active dispersal is initiated by the biofilm-resident cells themselves in response to environmental stressors such as antimicrobial exposure, matrix-degrading enzymes, and nutrient depletion; (ii) passive detachment occurs due to external physical forces such as shear forces or fluid flow [33,34,35]. Active dispersal typically results in the rapid release of planktonic cells or microcolonies, through mechanisms collectively referred to as follows (Figure 2): (i) abrasion is a mechanical loss due to collision with particles in the surrounding liquid [36]; (ii) erosion is a gradual release of individual cells or small clusters from the biofilm matrix [37,38]; (iii) sloughing is the sudden detachment of large biofilm sections, often caused by oxygen or nutrient depletion at the base of the biofilm or abrupt increases in nutrient availability in the surrounding liquid [38].

During active dispersal, bacteria typically upregulate genes involved in motility (e.g., flagella synthesis) and EPS degradation, while downregulating genes responsible for EPS production, surface attachment, and fimbriae synthesis [39]. Another regulatory mechanism involves c-di-GMP signaling. Inhibiting c-di-GMP pathways or reducing intracellular c-di-GMP levels can suppress biofilm formation and stimulate dispersal [31]. For example, oxygen limitation can promote c-di-GMP degradation, triggering detachment; glucose supplementation has been shown to reduce intracellular c-di-GMP, leading to increased flagella expression and enhanced dispersal capacity [35]. In addition to environmental cues, several physicochemical parameters and intrinsic bacterial properties, such as autolysis, also contribute to biofilm dispersal [35,39].

## 2. Clinical Problem of Biofilm

### 2.1. Biofilm-Mediated Immune Evasion and Virulence Strategies

A major clinically relevant challenge posed by biofilms is the ability of the bacteria within them to evade the host immune system. Roilides et al. [40] reported that biofilm-associated bacteria achieve immune evasion by reducing their metabolic activity, altering antigen expression, and secreting immunosuppressive molecules. A clear example of this mechanism is provided by the Gram-positive strain *Staphylococcus aureus*. According to Merino et al. [41], the expression of *S. aureus* protein A (SpA) enhances bacterial aggregation and biofilm formation. SpA not only promotes aggregation but also contributes to immune evasion by binding to the Fc region of IgG antibodies, thereby inhibiting opsonization and complement activation, two key processes of the innate immune response. In addition, SpA functions as a superantigen, simultaneously activating T and B lymphocytes, which ultimately leads to their depletion. This mechanism impairs the adaptive immune response and prevents the development of protective antibodies [42]. The combined effects of antibody interference and superantigen activity enable *S. aureus* to evade both the humoral (antibody-mediated) and cellular (T-cell-mediated) branches of the adaptive immune system [42].

Moreover, *S. aureus* secretes several cytotoxic molecules that contribute to tissue damage and bacterial dissemination. Among these are leucocidins, such as Panton-Valentine leucocidin (PVL), which specifically target and lyse neutrophils, weakening the host’s first line of defense, and hemolysins. *S. aureus* produces four types of hemolysins—alpha, beta, gamma, and delta—that disrupt host cell membranes, promoting immune evasion and tissue invasion [43,44].

Generally, biofilms are not composed of a single species but are predominantly multi-species communities. Interspecies cooperation within biofilms can enhance virulence by enabling different microbial species to produce toxins or enzymes that they could not generate individually, or by increasing the effectiveness of existing virulence factors [45].

### 2.2. Multifactorial Antibiotic Resistance in Biofilm Communities

The second major clinically relevant issue associated with biofilms is their strong resistance to antibiotics conferred by physical barriers, physiological factors, and genetic mechanisms.

The extremely low permeability of the EPS, estimated to range between 10^−18^ and 10^−15^ m^2^ [46], creates a physical barrier that impedes or significantly slows the penetration and diffusion of antibiotics within the biofilm.

Moreover, the high bacterial density in biofilms can increase the frequency of genetic mutations and facilitate the horizontal transfer of antibiotic resistance genes through mechanisms such as conjugation. The presence of efflux pumps within the biofilm further contributes to resistance by actively reducing intracellular antibiotic concentrations to sub-lethal levels, thereby promoting the selection and persistence of resistant strains [47].

Physiological heterogeneity within the biofilm also plays a crucial role. Variations in nutrient and oxygen availability lead to metabolic diversification, with some bacteria entering a dormant or low-metabolic state that makes them less susceptible to antibiotic treatment. This metabolic dormancy is accompanied by significantly slower growth rates compared to planktonic (free-floating) bacteria. Since many antibiotics are most effective against actively dividing cells, their efficacy is markedly diminished in the biofilm environment [48]. The presence of these dormant cells poses an additional clinical challenge, as they can survive antibiotic treatment and later reawaken when conditions become favorable, leading to recurrent and persistent infections.

Bacterial biofilms pose a serious clinical problem, accounting for 65–80% of all human infections, both chronic and acute. They are accountable for chronic inflammation, impaired healing, and recurrent infections. Biofilms can develop on both biological tissues and medical devices (e.g., prostheses, catheters, valves), and device-associated infections contribute considerably to disease morbidity, mortality, and healthcare expenses [49].

### 2.3. Medical Device-Associated Infections

The implantation of a prosthesis creates an opportunity for bacterial infection and the subsequent development of a bacterial biofilm, especially in individuals with an impaired immune system [50]. These infections are relatively common and are associated with a significant increase in morbidity, mortality, and healthcare costs. Stewart et al. indicate that approximately 5% to 10% of orthopedic implants become infected [51]. *Staphylococcus aureus* and *Staphylococcus epidermidis* are the predominant causative agents, followed by other Staphylococcal species such as *S. hominis* and *S. haemolyticus*, as well as Gram-negative strains like *Pseudomonas aeruginosa* and *Enterococcus faecalis* [52,53].

Trampuz and Zimmerli categorized the infections by the time of onset after implantation [54]: (i) early infections (first three months) are generally caused by highly virulent organisms (e.g., *S. aureus*); (ii) delayed infections (3–24 months post-surgery) are often due to less virulent microbes; (iii) late or secondary infections (>24 months), often stemming from infections in adjacent tissues or organs. These infections can occur at various sites, with the most life-threatening and clinically significant being infections of prosthetic hips; catheter-associated infections; central venous catheter (CVC) infections; ventilator-associated pneumonia; and prosthetic valve infections.

#### 2.3.1. Infections of Prosthetic Hips

Infections of prosthetic hips must be taken seriously because of the potential for serious complications. The incidence has been variously reported to range between 0.5% and 2%, with a 1-year mortality rate estimated at 4.22% according to a meta-analysis [55,56]. Older patients, especially those with pre-existing conditions, like cardiovascular diseases, diabetes mellitus, and cancers, or autoimmune diseases like rheumatoid arthritis, are at increased risk. The treatment of such cases is often complex and may even require the removal of the implant, thereby compromising the success of the intervention. *Staphylococcus* spp. are always the most frequently identified pathogens in such cases.

#### 2.3.2. Catheter-Associated Infections

Catheter-associated infections are one of the most prevalent device-associated infections. Urinary catheters are used in approximately 25% of hospitalized patients at some point during their stay, making catheter-associated urinary tract infections (CAUTIs) a significant cause of morbidity, mortality, and prolonged hospital stays [57]. According to the International Society for Infectious Diseases, about 5% of patients with CAUTIs develop bacteremia, which carries a mortality rate of around 30% [58]. The causative organisms are typically part of the patient’s endogenous flora or are introduced via the hands of healthcare workers. The most frequently implicated pathogens include *Escherichia coli*, *Pseudomonas* spp., *Klebsiella* spp., *Enterobacter* spp., and *Candida* spp.

#### 2.3.3. Central Venous Catheters (CVCs)

Central venous catheters (CVCs), particularly in intensive care units, are a considerable source of hospital-acquired infection [59]. Guggenbichler et al. [60] estimated that nearly 250,000 patients in the United States are infected every year, and the mortality rate associated with this can be as high as 35%. *S. epidermidis*, *S. aureus*, *Enterococcus* spp., *Candida* spp., *P. aeruginosa*, and *Klebsiella pneumoniae* are the most frequently isolated microorganisms in CVC infections.

#### 2.3.4. Ventilator-Associated Pneumonia (VAP)

Ventilator-associated pneumonia (VAP) is one of the most common hospital-acquired infections in intensive care units (ICUs), typically occurring after 48 h of mechanical ventilation via tracheal intubation [61]. It affects 20–36% of ventilated patients and is mainly caused by the colonization of respiratory devices by Gram-negative bacteria from the oral or gut microbiota [62]. VAP is a major contributor to morbidity, prolonged hospital stays, increased healthcare costs, and has a mortality rate ranging from 27% to 76%, especially in surgical ICUs [63]. Late-onset VAP is often associated with multidrug-resistant pathogens, such as methicillin-resistant *S. aureus* (MRSA), *P. aeruginosa*, *Acinetobacter* spp., *Enterobacter* spp., *Klebsiella* spp., and *Serratia* spp. [64]. Patients with invasive respiratory devices, such as endotracheal tubes or tracheostomies, are at particularly high risk.

#### 2.3.5. Prosthetic Valve Infections

Although less frequent, infections of prosthetic heart valves are particularly severe and can be associated with mortality rates reaching 19–50% [65]. These infections, known as prosthetic valve endocarditis, typically arise from microbial colonization of the valve surface, followed by the development of a biofilm. The incidence is estimated at around 4–5% for arterio-venous and femoro-popliteal grafts. Although aortic graft infections are rarer—occurring in about 2% of cases—they are associated with a particularly high mortality rate, reaching up to 75% [66]. The most commonly involved microorganisms include *S. epidermidis*, *S. aureus*, *Streptococcus* species, Gram-negative bacilli, *Enterococcus* spp., and *Candida* spp.

## 3. Impact of Extracellular Polymeric Substance on Biofilm Formation

Biofilm is a heterogeneous structure composed primarily of water (85–95%), microbial cells (10–25% of dry mass), and a self-produced EPS matrix (75–90% of dry mass), which includes polysaccharides, proteins, lipids, DNA, and RNA [2]. In this complex architecture, the interstitial voids or water channels within the biofilm are essential for separating microcolonies [67]. The EPS forms a shield that holds the biofilm together, facilitating cell-to-cell communication and providing the adhesion and cohesion necessary for biofilm formation. Moreover, EPS contributes to nutrient cycling, maintains the availability of DNA for horizontal gene transfer, and acts as a protective barrier against oxidizing biocides, antibiotics, ultraviolet radiation, desiccation, and host immune defenses [68].

**Figure 3:**
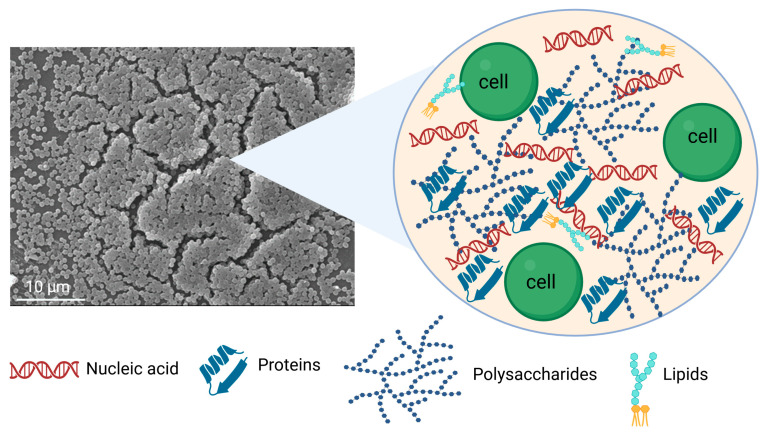
Main components in the EPS matrix of biofilm (created in BioRender. Cochis, A. (2025) https://BioRender.com/g2n7f8z, accessed on 1 August 2025).

The main components of EPS can be categorized as follows (Figure 3):

### 3.1. Water

Water is the largest component of the EPS matrix in biofilms. It maintains hydration and protects the biofilm from desiccation, even under fluctuating environmental moisture conditions. The availability and distribution of water directly influence the flow and maintenance of essential nutrients within the biofilm [69]. Furthermore, water-filled channels enable the transport of nutrients, waste products, signaling molecules, and antibiotics through the biofilm [67].

### 3.2. Polysaccharides

The majority of biofilms depend on secreted extracellular polysaccharides, which constitute a primary component of the EPS matrix [70]. These polysaccharides can be categorized into homopolysaccharides and heteropolysaccharides based on their monosaccharide composition. Most are heterogeneous; however, some are homogeneous, such as fructan in *Actinomycetes naeslundii* [71], glucan in *Streptococcus mutans*, and β-glucan in *Lactobacillus* spp. [72]. In *Escherichia coli*, three major heteropolysaccharide ESP components have been identified: poly-N-acetyl-glucosamine (PGA), colanic acid (CA), and cellulose. PGA is considered crucial for biofilm formation and is found in various eubacterial species [73]. Colanic acid, composed of glucose, galactose, fructose, and glucuronic acid, is prevalent among *E. coli* isolates and is thought to play a role in bacterial survival outside the host [74]. Although cellulose is often the most abundant EPS component in many *E. coli* strains, it is absent in the K-12 strain due to a single-nucleotide polymorphism in the *bcsQ* gene [75]. Many clinically significant bacterial pathogens produce a common exopolysaccharide known as partially de-N-acetylated poly β-(1,6)-N-acetyl-d-glucosamine (dPNAG) as a key biofilm matrix component [76]. Both Gram-positive and Gram-negative bacteria produce dPNAG (referred to as polysaccharide intercellular adhesin in bacterial strains), including *Staphylococcus aureus*, *Escherichia coli*, *Aggregatibacter actinomycetemcomitans*, *Acinetobacter baumannii*, and *Klebsiella pneumoniae* [76]. In *Pseudomonas aeruginosa*, three main exopolysaccharides, Pel, Psl, and alginate, play central roles in biofilm formation and architecture. Pel is a cationic polysaccharide containing acetylgalactosamine and acetylglucosamine. It facilitates cell–cell adhesion, surface attachment, DNA cross-linking, and protection against aminoglycoside antibiotics [77,78]. Psl is rich in mannose, glucose, and rhamnose and is involved in both cell–cell interactions and surface attachment [77,79]. Alginate, a mucoid polysaccharide composed of guluronic and mannuronic acids, is commonly found in *P. aeruginosa* isolates from cystic fibrosis (CF) lung infections. It contributes to biofilm formation and immune evasion but plays a limited role outside the CF lung environment [77,80].

### 3.3. Proteins

Extracellular proteins in the biofilm matrix include secreted enzymes, structural proteins, subunits of surface appendages (e.g., pili and flagella), surface adhesins, and proteins contained in outer membrane vesicles (OMVs) [70]. These proteins interact with exopolysaccharides and nucleic acids, contributing to matrix stabilization, surface colonization, and the overall structural integrity of the biofilm [81]. Under nutrient-limited conditions, bacteria secrete enzymes capable of degrading EPS components to access alternative energy sources [70]. Examples include glycoside hydrolases such as dispersin B (DspB), which breaks down polysaccharides [82], and DNases, which degrade extracellular nucleic acids [83]. The impact of these enzymes on the EPS matrix will be discussed in more detail in the following sections. Jiao et al. [84] reported a significant difference between the proteome composition of EPS and that of individual cell fractions, identifying a high concentration of protein peptidases, cell wall, polysaccharide metabolism enzymes, disulfide-isomerases, and chaperones such as cold shock and DNA-binding proteins in the EPS matrix. Vo et al. [85] showed that a major part of cytosolic proteins, including anionic ribosomal proteins, is closely bound to poly-(1→6)-β-N-acetylglucosamine (PNAG) in live *S. epidermidis* biofilms. These proteins are presumably released during cell lysis, which occurs as part of biofilm maturation, and remain bound to the cationic PNAG within the EPS. Notably, they found that the well-known *S. epidermidis* biofilm protein accumulation-associated protein (Aap) does not directly interact with PNAG. Importantly, they identified elastin-binding protein (EbpS), a microbial surface component involved in adhesion to human elastin peptides, as being highly enriched in a PNAG-dependent manner in *S. epidermidis* biofilms. Toyofuku et al. [86] demonstrated that approximately 30% of the EPS matrix proteins in *P. aeruginosa* are membrane-associated and found within OMVs, while other proteins originate from lysed cells or are secreted.

### 3.4. Nucleic Acids

Extracellular DNA (eDNA) is a key component of the EPS matrix, playing an important role in microbial aggregation within the biofilm. The source of eDNA can vary; however, cell lysis is the primary mechanism in many bacterial species [81]. In *Streptococcus gordonii*, hydrogen peroxide-induced cell lysis contributes to eDNA release and subsequent EPS formation [87,88]. In *S. mutans*, eDNA may be released via membrane vesicle extrusion or (auto)lysis [88,89]. In human infections such as cystic fibrosis (CF), the eDNA found in *P. aeruginosa* biofilms originates primarily from human polymorphonuclear leukocytes (PMNs), which are recruited to the infection site [90]. EndA, a DNA-specific endonuclease, degrades eDNA within *P. aeruginosa* biofilms, leading to biofilm dispersal [91]. These findings align with recent reports indicating that eDNA is primarily responsible for the viscoelasticity of *P. aeruginosa* biofilms [92], with the loss of eDNA fiber structures coinciding with biofilm dissolution. However, the exact mechanism by which eDNA contributes to biofilm viscoelasticity and the nature of its supramolecular interactions remain unclear [93].

Extracellular RNA (eRNA) is also present in high concentrations in nucleic acid-containing biofilms and may contribute to eDNA-based viscoelastic networks [93]. Most established knowledge surrounds eDNA, but eRNA is gaining attention for its potential structural and regulatory roles in biofilms. It can be interactions and functional connections between eRNA and eDNA in biofilm matrices, though this is a relatively emerging area of research [94]. They have potential for DNA-RNA hybrid structures, although their stability and role in biofilms are not fully elucidated. Some studies suggest the eRNA can cooperate with eDNA in biofilm matrix stabilization and maintain the viscoelasticity, particularly in *P. aeruginosa* [93]. Additionally, in *S. aureus* biofilms, eRNA was reported to serve a structural function by associating with eDNA and stabilizing the polysaccharide-rich matrix [93]. Ribonuclease treatment has been shown to inhibit the formation of eDNA-containing Nontypeable Haemophilus influenzae (NTHI) biofilms [93]. Nevertheless, the lack of robust eRNA extraction protocols and the inherent stability of the molecule [95] have hindered a comprehensive understanding of its precise structural role or its potential interactions with eDNA in biofilm matrices [93].

### 3.5. Surfactants and Lipids

Lipid A, a key component of lipopolysaccharide (LPS) located in the outer membrane of Gram-negative bacteria, can also be found in the EPS of biofilms. In addition, several lipids with surface-active properties, such as surfactin, emulsan, and viscosin, are present in the EPS matrix. These biosurfactants enhance the availability of hydrophobic substances by dispersing them [96,97]. Ron and Rosenberg [98] reported that biosurfactants contribute to heavy metal binding and the production of virulence factors. Akter et al. [99] evaluated biofilm formation by *P. aeruginosa* and *E. coli* on various food-contact surface coupons, particularly aluminum (ALU), silicone rubber (SR), and polyethylene terephthalate (PET). The resulting biofilms exhibited a complex mixture of hydrophilic and hydrophobic components. As they matured, the polysaccharide structures remained stable while the lipid content increased, contributing to enhanced biofilm resilience.

## 4. Mitigation of Biofilm Formation on Biomaterials via EPS Matrix Disruption

The EPS matrix presents a major challenge for biofilm eradication strategies due to its chemical complexity and strain-specific variability [97]. Nonetheless, recent advances have led to the development of innovative approaches targeting this matrix. The following sections of this review focus on strategies to modify biomaterial surfaces to disrupt the EPS and thereby mitigate biofilm formation. The increasing resistance of bacteria to antibiotics, combined with the limited effectiveness of conventional antimicrobials against biofilm, has driven attention toward the use of non-antibiotic agents to modify surfaces to disturb the EPS matrix as a primary target.

### 4.1. Biofilm-Dispersing Enzymes

Biofilm-dispersing enzymes have demonstrated effectiveness against both developing and mature biofilms, typically requiring relatively low concentrations to achieve high specificity and efficacy. Unlike small-molecule drugs, which often encounter antibiotic resistance, these enzymes function extracellularly and do not require transport across the bacterial membrane, reducing the likelihood of resistance development. Three major classes of biofilm-disrupting enzymes have been extensively studied, namely glycoside hydrolases, proteases, and deoxyribonucleases (DNases). These enzymes enhance therapeutic outcomes by degrading key components of the EPS matrix, thereby improving antimicrobial penetration and immune cell access [76,100].

#### 4.1.1. Glycoside Hydrolysis Enzyme

Dispersin B (DspB) is a member of glycoside hydrolase family 20 (GH20) and was initially isolated from *Aggregatibacter actinomycetemcomitans* [76]. DspB hydrolyzes the exopolysaccharide dPNAG in biofilm matrices through both endo- and exo-glycosidic activity [101,102]. This enzyme has been shown to inhibit biofilm and pellicle formation, detach mature biofilms, disaggregate bacterial clusters, and increase the susceptibility of preformed biofilms to enzymatic removal [103]. Ghalsasi et al. [104] engineered *E. coli* K-12 W3110 to synthesize and secrete DspB, which enables the degradation of poly-N-acetyl glucosamine (PGA) and subsequent disruption of target biofilms. Liu et al. [105] utilized magnetoreceptor (MagR), a magnetic protein responsive to external magnetic fields, as a fusion partner for immobilizing DspB on magnetic Fe_3_O_4_@SiO_2_ core–shell particles, with Fe_3_O_4_ as a core and SiO_2_ as a shell. Pavlukhina et al. [106] developed a biocompatible surface-attached polymer matrix via layer-by-layer (LBL) deposition for DspB delivery. The LbL system was constructed using poly(allylamine hydrochloride) (PAH) and poly(methacrylic acid) (PMAA), followed by glutaraldehyde crosslinking and pH-triggered removal of PMAA, resulting in a stable PAH hydrogel suitable for DspB loading. This system achieved more than 98% biofilm inhibition for two clinical isolates of *S. epidermidis* compared to mock-loaded multilayers [106]. Although DspB does not possess intrinsic antimicrobial activity, it shows enhanced in vitro efficacy against biofilms when combined with agents such as silver, cefamandole nafate, and ampicillin [107,108]. By degrading PNAG, DspB also prevents biofilm formation by disrupting the structural scaffold of the EPS matrix. Kane Biotech (Winnipeg, MB, Canada), a Canadian company, has trademarked Dispersin B^®^ for the development of novel therapies targeting staphylococcal biofilms [82]. When combined with teicoplanin in catheter lock solutions, DspB dispersed *S. aureus* biofilms and improved the clearance of bloodstream infections in catheterized sheep [82]. It also showed efficacy in catheter-associated infections in rabbits when combined with triclosan [109]. However, these studies assessed only efficacy, without addressing toxicity or enzyme stability. Research has primarily focused on the use of DspB on Gram-positive bacteria, particularly staphylococci, dominant pathogens in skin and wound infections. Nonetheless, DspB could have broader applications in treating bacterial biofilms, and Kane Biotech (Winnipeg, MB, Canada) is also marketing Dispersin B^®^ as an oral care product for domestic animals.

PgaB is another glycoside hydrolase encoded by the pgaB gene of the PNAG biosynthetic operon. It is capable of degrading PNAG analogues and disrupting PNAG-dependent biofilms formed by *Bordetella pertussis*, *Staphylococcus carnosus*, *S. epidermidis*, and *E. coli* [110,111]. However, DspB and Pga B differ in their catalytic mechanism: DspB acts as both an exo- and endo-glycosidase, cleaving internal and terminal glycosidic bonds, whereas PgaB is strictly an endo-glycosidase (cleaves within a polysaccharide unit). In *S. epidermidis*, DspB exhibits a lower half-maximal effective concentration (EC50; 0.24 nM) compared to PgaB, although both enzymes enhance the activity of antimicrobials to a similar extent [110,112].

In addition to dPNAG-targeting enzymes, alginate lyases have proven effective in dispersing mature biofilms [113]. These enzymes, which degrade alginate, have been isolated from various organisms with different substrate specificities, including algae, marine mollusks, marine and terrestrial bacteria, and some viruses and fungi [114]. Alginate lyase treatment reduces viscosity in cultures and cystic fibrosis (CF) sputum, strips biofilms from abiotic surfaces, promotes *P. aeruginosa* phagocytosis by immune cells, and enhances the efficacy of antipseudomonal antibiotics [113]. Other notable glycoside hydrolases include PslG (GH39 family) and PelA (a periplasmic glycoside hydrolase), which inhibit biofilm formation by clinical and environmental *P. aeruginosa* isolates and disrupt newly formed biofilms over 24 h [115,116]. Chareza et al. [117] immobilized alginate lyase onto bacterial cellulose (BC) pellicle produced by *Komagataeibacter xylinus* via physical adsorption, protecting wounds from *P. aeruginosa* infection and significantly reducing biofilm polysaccharide content. Wan et al. [118] demonstrated pH-responsive drug release using silver nanocomposites co-delivering alginate lyase and ceftazidime, effectively inhibiting and degrading *P. aeruginosa* PAO1 biofilms. Implanted medical devices, such as central venous catheters, are highly susceptible to microbial colonization and biofilm formation and represent a significant risk factor for nosocomial infections. *P. aeruginosa* relies on exopolysaccharides like Psl for initial attachment and biofilm maturation. Asker et al. [99] immobilized Psl-specific glycoside hydrolase (PslGh) onto the lumen surfaces of polyethylene, polyurethane, and polydimethylsiloxane (silicone) catheters. Under dynamic in vitro flow conditions, these modified catheters showed a 3-log reduction in bacterial counts over 11 days and a 2-log reduction by day 14, compared to untreated catheters as controls. In an in vivo rat model, PslGh-coated PE-100 catheters demonstrated a ~1.5-log reduction in colonization by the clinical *P. aeruginosa* ATCC 27,853 strain after 24 h [119]. Table 1 summarizes other well-characterized glycoside hydrolases with established biofilm-dispersing properties.

#### 4.1.2. Proteases

Exoproteins, another major component of the EPS, account for a significant portion of the biomass in most biofilms. They play critical roles in bacterial aggregation, surface adhesion, and maintaining the structural integrity of biofilm matrices [140]. Enzymatic degradation of EPS exoproteins is one of the most effective strategies for biofilm eradication. To date, a variety of proteases with biofilm-dispersing capabilities have been identified and studied. These include Proteinase K, trypsin, pepsin, aureolysin, Spl proteases, and staphopain A and B, which are produced by various microorganisms to degrade biofilms [103,141].

Proteinase K is a broad-spectrum serine protease that cleaves peptide bonds near carboxylic groups of amino acids. It inhibits *S. aureus* biofilm formation by disrupting early adhesion and is capable of dispersing biofilms aged 24 and 48 h [103]. Although direct covalent attachment of proteinase K to biomaterial surfaces is less commonly reported, its incorporation into coatings or delivery systems has been explored. For instance, Sun et al. [142] developed multifunctional nanohybrids by combining phenylboronic acid-modified carbon dots (PCDs) with proteinase K, enhancing photosensitization to overcome biofilm barriers. In vitro studies demonstrated that PCDs were effective against a variety of bacteria, including multidrug-resistant strains, and exhibited strong affinity for both Gram-positive and Gram-negative species. This resulted in elevated reactive oxygen species (ROS) levels and, when combined with proteinase K, facilitated deep penetration and active biofilm elimination, improving treatment outcomes [142].

Trypsin, a pancreatic serine protease that cleaves peptide bonds on the carboxyl side of lysine and arginine residues, has been used to disrupt biofilms on teeth and wound surfaces [143,144]. Bovine trypsin has demonstrated the ability to degrade mature biofilms of various Gram-positive and Gram-negative bacteria [143]. It can reduce the biomass of 24 h old biofilms of *P. aeruginosa* and *Enterococcus faecalis*, though it does not completely eradicate them, regardless of treatment duration or enzyme concentrations [145]. Caro et al. [146] proposed a promising antibiofilm strategy by covalently immobilizing lysozyme and trypsin on stainless-steel oxide surfaces to inhibit biofilm formation.

Pepsin is a broad-specificity endopeptidase that contains a catalytic aspartate residue in its active site and preferentially cleaves at phenylalanine and leucine residues, but its activity is hindered by histidine, lysine, arginine, and proline residues [147]. Like trypsin, pepsin reduces the biomass of 24 h old biofilms of *P. aeruginosa* and *E. faecalis*, but it cannot fully remove biofilms from polystyrene surfaces, regardless of enzyme concentrations or exposure time [145]. However, when co-administered with trypsin and carvacrol, it has been shown to effectively eradicate preformed *P. aeruginosa* and *E. faecalis* biofilms [148]. Table 1 summarizes other proteolytic enzymes known for their biofilm-dispersing activity.

#### 4.1.3. Deoxyribonucleases

eDNA is a ubiquitous and essential structural component of the EPS, contributing to microbial adhesion, cell signaling, horizontal gene transfer, and biofilm matrix stabilization [149,150]. Although eDNA plays a vital role in biofilms, its importance went largely unrecognized until 2002, when Whitchurch et al. [151] demonstrated that exogenous addition of DNase I could disperse biofilms and enhance the bactericidal activity of antibiotics. Since then, considerable research has focused on using various DNases to target eDNA for biofilm eradication.

DNase I is a widely studied pancreatic endonuclease that specifically digests DNA and has been shown to disrupt biofilm formation in both mono- and polymicrobial communities [22]. However, DNase I is more effective against early-stage biofilms. It can degrade newly formed biofilms (up to 60 h old), whereas mature biofilms (older than 84 h) display considerable resistance to DNase I. This resistance is likely due to the structural reinforcement of the biofilm by other EPS components such as exopolysaccharides and exoproteins. Additionally, mature biofilms may produce proteolytic exoenzymes that locally inactivate DNase I [151]. Recombinant human DNase I (rhDNase) has been clinically applied to reduce the viscosity of purulent sputum in patients with cystic fibrosis [152]. Ye et al. [153] demonstrated that immobilization of DNase I on titanium (Ti) surfaces using dopamine as a crosslinking agent significantly reduced the adhesion and biofilm formation of *S. mutans* and *S. aureus* over 24 h. Aktan et al. [154] evaluated the effectiveness of alternating current electrophoretic deposition (AC-EPD) as a novel method to apply DNase I onto titanium surfaces. Their results showed a significant reduction in biofilm formation by *S. epidermidis* and *P. aeruginosa* up to 20 h post-treatment. Dornase alfa, a recombinant human deoxyribonuclease I (rhDNase I), has been approved by the Food and Drug Administration (FDA) under the brand name Pulmozyme^®^. Daily inhalation of Dornase alfa has been shown to reduce the viscoelasticity of sputum, lower the frequency of pulmonary exacerbations, and improve lung function in patients with cystic fibrosis [152]. Table 1 summarizes other deoxyribonuclease enzymes known for their biofilm-dispersing activity.

### 4.2. Chelating Agents

Metallic cations, like Fe^3+^, Mg^2+^, and Ca^2+^, are essential to microbial adherence, biofilm formation, and bacterial growth [155,156]. Recently, many efforts have been made to utilize high-affinity metal-binding agents to chelate these ions, inhibiting bacterial growth. The most common chelating agents are Ethylenediamine-tetra-acetic acid (EDTA), Trisodium citrate (TSCs), and Ethylene glycol-bis (β-aminoethyl ether)-N, N, N’, N’-tetra-acetic acid (EGTA).

EDTA is a metal chelator normally used in clinics as an anticoagulant agent. However, in the early 2000s, clinicians saw an inhibitory activity of EDTA against methicillin-resistant Staphylococci, Gram-negative bacilli, and Candida species. EDTA can efficiently bind iron, magnesium, and calcium required to stabilize the extracellular biofilm matrix [157]. The biofilm matrix destabilization not only enhances the detachment of bacteria from a surface but may also increase the bacterial cell permeability and help the penetration of antibiotics such as gentamicin or cefotaxime [158,159].

Similar to EDTA, TSC chelates Ca^2+^ and Mg^2+^ and inhibits biofilm formation. Chu et al. [160] demonstrated that a 4% TSC solution can prevent biofilm formation by *S. aureus*, *P. aeruginosa*, *E. coli*, and *K. pneumoniae*. Unlike EDTA, TSC also inhibits extracellular matrix production and quorum sensing.

EGTA is a chelating agent with a much higher affinity for Ca^2+^ ions than for Mg^2+^ ions, making it useful for selectively binding calcium in the presence of magnesium. Lyamba et al. [161] demonstrated that low concentrations of EGTA prevent the adhesion of *S. aureus* to abiotic surfaces and inhibit biofilm development. Furthermore, once established, the biofilm becomes resistant to EGTA, suggesting that EGTA-containing lock solutions should be used to prevent biofilm formation rather than to promote its removal.

In addition to EDTA, TSC, and EGTA, many other chelators are effective in inhibiting bacterial adhesion and biofilm formation. The most recently studied include transferrin, lactoferrin, desferrioxamine (DFO), deferiprone (DFP), and kojic acid [162,163]. DFP and kojic acid have been shown to inhibit *P. aeruginosa* biofilm formation without affecting bacterial growth [164]. However, higher concentrations of DFO may promote biofilm formation by acting as an iron carrier.

### 4.3. Quorum Sensing Inhibitors

**Figure 4:**
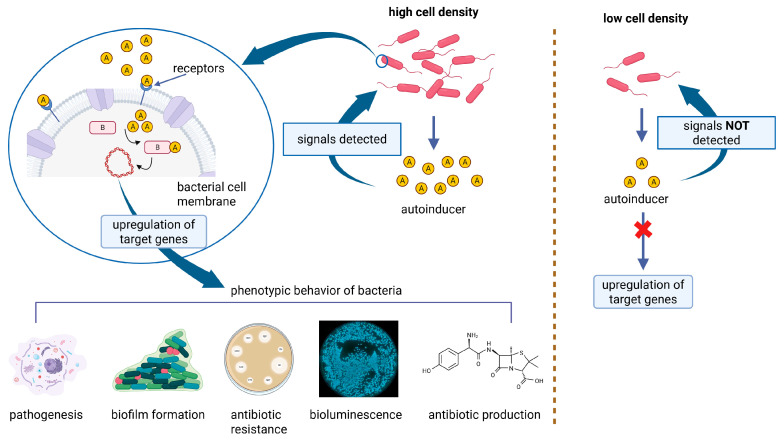
Schematic explanation of the QS system in bacterial strains that regulates virulent behaviors (created in BioRender. Cochis, A. (2025) https://BioRender.com/g4wg72q, accessed on 1 August 2025).

The development of bacterial biofilms involves self-organization, cooperation, and communication among microbial communities as they transition from free-floating planktonic cells to a structured, three-dimensional biofilm lifestyle. Multiple environmental cues, including stress, nutrient limitation, and intercellular communication, influence this transition. To adapt to changing environments, bacteria rely on coordinated gene expression triggered by these external stimuli [165]. A key mechanism facilitating this coordination is quorum sensing (QS), a form of bacterial cell-to-cell communication that regulates gene expression based on population density. QS operates through the release, detection, and uptake of small diffusible signaling molecules known as autoinducers (AIs). These molecules accumulate in the environment and, once a threshold concentration is reached, trigger a synchronized response among bacterial cells. QS can occur within a single species (intraspecies) or between different species (interspecies) and regulates several physiological and pathogenic behaviors, including horizontal gene transfer, expression of virulence factors, synthesis of secondary metabolites (e.g., antibiotics), motility, and biofilm formation (Figure 4) [166]. The QS systems of many Gram-positive and Gram-negative bacteria are well characterized. In Gram-negative species, the primary AIs include N-acyl homoserine lactones (AHL), autoinducer 2 (AI-2), a furanosyl borate diester, and autoinducer 3 (AI-3). QS plays a critical role in various stages of biofilm formation by modulating processes, such as bacterial motility, adhesin production, extracellular polysaccharide synthesis, and the release of eDNA [167]. These factors contribute to both biofilm maturation and cell dispersion, enabling the biofilm to maintain structural integrity while ensuring the ability of cells to detach and colonize new environments. Through these regulatory pathways, QS enhances the adaptability, persistence, and pathogenic potential of bacterial communities [5]. Guendouze et al. reported the effectiveness of the lactonase enzyme SsoPox-W2631 in attenuating the virulence of 51 clinical *P. aeruginosa* isolates obtained from diabetic foot ulcers. This enzyme significantly reduced the secretion of proteases and pyocyanin, leading to a subsequent decrease in biofilm formation [168]. Vogel et al. immobilized the quorum-quenching enzyme PvdQ, an N-terminal nucleophile acylase encoded within the pyoverdine (pvd) gene cluster, onto polydimethylsiloxane silicone (PDMS) surfaces. This functionalized coating demonstrated quorum-quenching activity and effectively reduced bacterial colonization on indwelling medical devices such as urinary and intravascular catheters [169].

### 4.4. Biosurfactants

Biosurfactants are biologically synthesized microbial products that have gained worldwide attention owing to their eco-friendly, biodegradable characteristics and their immense effectiveness in bioremediation, pharmaceuticals, agriculture, the food industry, and anti-biofouling [170]. Some studies have revealed that the inhibition of bacterial adhesion and biofilm dispersal properties of the biosurfactants is due to their surface and interfacial tension reduction, as well as solubilized lipids and proteins in the EPS matrix, interruption in the QS signaling system, and an increase in the bacterial membrane permeabilization [171,172,173]. Sophrolipids, produced by *Starmerella bombicola* (previously classified as *Candida bombicola*), showed bactericidal properties when compared to conventional antimicrobial agents with bacteriostatic effects [174]. Ceresa et al. [175] used this property of the sophrolipids and evaluated their antimicrobial properties in medical-grade silicon discs against several clinically relevant strains: *C. albicans*, *S. aureus*, and *P. aeruginosa*. The obtained results demonstrated that pre-coating silicone discs with lactonic sophrolipids (SLA) reduced *S. aureus* and *P. aeruginosa* by about 70–80% and inhibited bacterial attachment up to 95% [175].

For the production of nanoparticles, it is currently necessary to identify non-toxic technologies. The use of biosurfactants as coupling agents is a recent method for the sustainable, environmentally friendly, and compatible synthesis of bioactive metallic nanoparticles. Top-down strategies using biosurfactants as capping and stabilizing agents have been proposed in many studies, disclosing the fabrication of metallic nanocomposites, including Ag, Au, Cu, Pt, Pd, NiO, and ZnS, to limit or mitigate aggregation after and during the fabrication of the preferred nanocomposite [176]. Microbial and biosurfactant technologies for nanomaterial fabrication are still being developed because they are clean, safe, and environmentally friendly. Additionally, the self-assembly of amphiphilic molecules acting as surfactants is a facile method to produce organic nanoparticles [177] and can be used as stabilizers and help to maintain a regular shape because of the electrostatic attraction forces [178]. Farias et al. [178] prepared silver nanoparticles with a size of 1.13 nm using the biosurfactant of *P. aeruginosa* acting as a stabilizer. Khalid et al. [179] demonstrated a simple approach for the preparation of rhamnolipid (RL)-coated iron NP and proposed a synergistic antibacterial and antiadhesive mechanism against *P. aeruginosa* and *S. aureus* biofilms. Reactive oxygen compounds are mechanically generated by RL-coated iron NP (48 nm), which supports the antibacterial effect.

### 4.5. Oxidizing Agents

Oxidizing agents, also known as reactive species, are low-molecular-weight molecules that induce oxidation by accepting electrons from other substances. These molecules are highly unstable due to the presence of unpaired or missing electrons. Their accumulation within cells can lead to oxidative stress. When applied to biomaterials, oxidizing agents can help prevent biofilm formation by disrupting the bacterial redox balance and inducing oxidative stress, ultimately damaging or killing bacterial cells. The most common producers of reactive species include metal nanoparticles, graphene oxide coatings, nitric oxide (NO)-releasing biomaterials, ozone-based coatings, and halogens.

#### 4.5.1. Metal Ions and Metal Nanoparticles

Silver, gold, copper, zinc, and gallium-based ions, along with metal and metal oxide nanoparticles such as ZnO and MgO, have been extensively studied for their potent antibacterial activity against both planktonic bacteria and biofilms. These systems exert antimicrobial effects through multiple mechanisms, including disruption of bacterial adhesion, damage to the cell membrane, induction of oxidative stress, interference with QS, and enhancement in antibiotic efficacy via synergistic interactions. Additionally, metal-based nanoparticles release ions that disrupt bacterial membranes and interfere with vital cellular processes, thereby preventing biofilm formation. Moreover, they can contribute to biofilm eradication by activating the host immune response [180,181].

Ferraris et al. developed a titanium alloy coated with a zirconia matrix doped with silver for use in temporary fixation devices. The authors demonstrated that this coating effectively inhibits the initial adhesion of *S. aureus*, thereby preventing biofilm formation. This anti-adhesive effect is attributed not only to the antimicrobial properties of the zirconia–silver coating but also to the synergistic interaction with the surface nanotopography (<0.2 μm), which limits the availability of anchorage points for bacterial attachment [182].

Qais et al. developed gold nanoparticles (AuNPs-CA) bio-fabricated using an aqueous extract of Capsicum annuum and tested them against *P. aeruginosa* and *Serratia marcescens*. The results demonstrated that these nanoparticles not only reduced the virulence factors of *P. aeruginosa*, including pyocyanin production, elastase activity, and rhamnolipid synthesis, but also inhibited biofilm formation and EPS production in both bacterial species [183].

**Figure 5:**
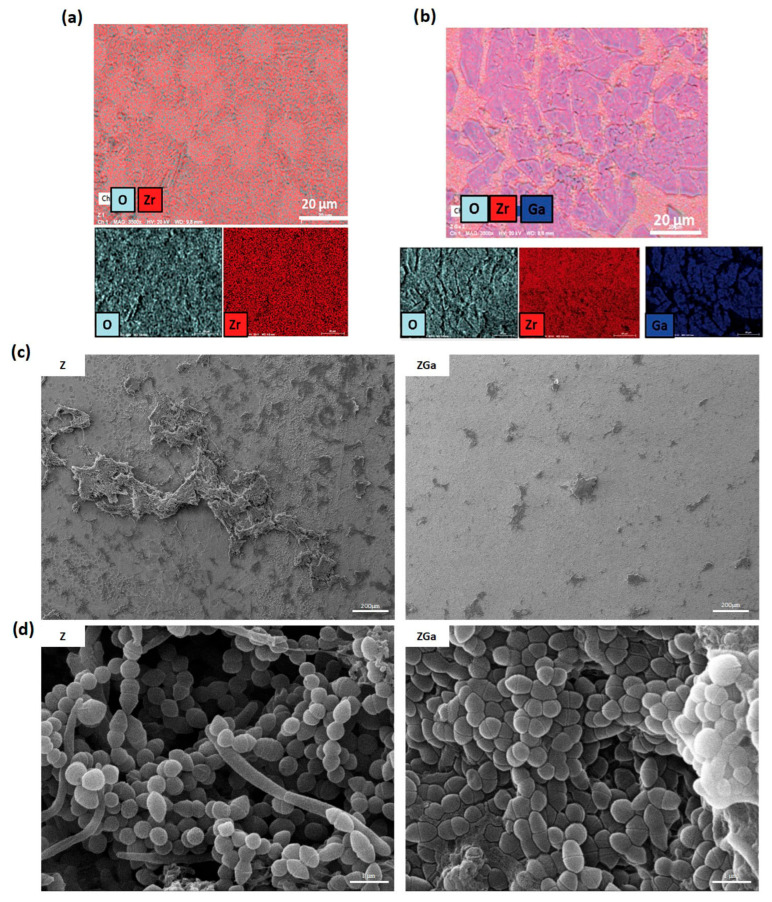
Gallium-doped zirconia reduces bacterial adhesion and proliferation. (**a**,**b**) Energy-dispersive X-ray spectroscopy (EDS) of zirconia (Z) and gallium-doped zirconia (ZGa); (**c**) low-magnification SEM micrographs showing a large bacterial biomass on Z and an almost clean surface on ZGa (scale bar = 200 μm); (**d**) high-magnification SEM micrographs highlighting the distinct bacterial populations on the two materials (scale bar = 1 μm). Reprinted/adapted with permission from [185], 2025, Alessandro Calogero Scalia.

Gallium (Ga) has garnered significant research interest over the past decade. Unlike many other metal ions, gallium is generally considered cytocompatible and exhibits selective antibacterial activity against pathogenic bacteria. In its ionic form (Ga^3+^), gallium exerts its antimicrobial effects by interfering with bacterial iron metabolism. This “Trojan horse” mechanism exploits bacterial iron uptake pathways to facilitate Ga^3+^ entry into the cell, where it disrupts iron-dependent enzymatic processes, ultimately inhibiting bacterial growth [184]. Recently, D’Agostino et al. [185] developed a gallium-doped zirconia coating for dental implant surfaces. The results showed that gallium selectively eliminates pathogenic bacteria while preserving commensal microbiota. Additionally, the coating significantly reduced oral plaque adhesion and overall biofilm biomass. Proteomic analysis of plaque samples in contact with the gallium-doped surface revealed a marked reduction in the adhesion of *Fusobacterium nucleatum*, a Gram-negative bacterium known for its role in oral biofilm stability and for shielding less aerotolerant species, such as *Porphyromonas gingivalis*, a key pathogen in periodontal disease, from oxidative stress (Figure 5).

The use of nanoparticles still raises several concerns related to toxicity. Their small size and large surface area can promote interactions with biological systems, potentially leading to adverse effects, such as oxidative stress, inflammation, DNA damage, immune system alterations, and organ toxicity [186,187]. To mitigate these risks, multifaceted strategies are required. Jangid et al. [188] highlighted that encapsulation technologies using biodegradable polymers can reduce silver nanoparticle (AgNP) toxicity without compromising their antimicrobial activity. Similarly, Rashid et al. [189] proposed that incorporating AgNPs into polymer matrices to create AgNP–polymer nanocomposites (AgNP–PNCs) allows for a controlled and sustained release of silver ions, thereby reducing the cytotoxicity typically associated with free AgNPs.

Another strategy to minimize nanoparticle-induced toxicity involves modulation of their size and shape. Blanco et al. [190] reported that nanoparticles smaller than 5 nm tend not to accumulate in vital organs and are efficiently cleared by the kidneys.

As with many innovative medical technologies, regulatory barriers and scalability represent major challenges. Nadar et al. [191] emphasized that nanoparticles face significant hurdles in the approval process, primarily due to the absence of standardized manufacturing and characterization protocols, inconsistent preclinical toxicity data, and a limited body of clinical evidence. In addition, the lack of long-term environmental impact assessments further complicates the regulatory approval pathway for nanoparticle-based products.

#### 4.5.2. Graphene Oxide

Graphene and graphene oxide (GO) coatings have emerged as promising antibacterial materials due to their ability to generate reactive oxygen species (ROS), which can damage bacterial cells and inhibit biofilm formation. In a study by Wang et al. [192], graphene was synthesized directly on the surface of a titanium alloy. The resulting graphene-coated titanium significantly reduced the adhesion and biofilm formation of *P. aeruginosa*, *F. nucleatum*, and *C. albicans*. Beyond their intrinsic antimicrobial properties, graphene-based materials also offer advantages as drug delivery platforms, thanks to their large surface area. Sun et al. [193] demonstrated that graphene not only kills pathogenic bacteria and prevents biofilm development but also enables the controlled release of levofloxacin, enhancing its antibacterial activity against *S. aureus* and *E. coli*. Moreover, Cheng et al. [194] showed that a graphene oxide coating can efficiently carry and release the antimicrobial peptide NAL-P-113, effectively targeting peri-implant pathogens such as *S. mutans* and *P. gingivalis*.

#### 4.5.3. Nitric Oxide

Nitric oxide (NO)-releasing materials can hinder biofilm formation by depleting exopolysaccharides and damaging bacterial membranes. Sadrearhami et al. [195] developed an antibiofilm coating that contains a sulfur-nitroso thiol NO donor and can produce adjustable NO fluxes. The plasma coatings were shown to retain a high percentage of sulfur groups, and their thickness could be predictably adjusted through deposition time. Coatings with a 250 nm film thickness reduced *P. aeruginosa* biofilm formation by 75% after 6 h and 55% after 24 h of exposure. Increasing the polymer coating thickness to 500 nm improved antibiofilm efficiency, achieving 81% and 52% inhibition of bacterial attachment after 24 and 36 h of incubation in bacterial culture solution, respectively.

Chug et al. [196] developed a portable NO-releasing catheter insert that combines antimicrobial effectiveness, biocompatibility, and ease of activation. The system employs a light-sensitive NO donor, S-nitroso-N-acetylpenicillamine (SNAP), which is covalently bonded to a polydimethylsiloxane (PDMS) matrix. This covalent attachment enhances NO retention and minimizes early leaching. The resulting SNAP-PDMS composite was applied to a side-emitting optical fiber and integrated with a wearable light-emitting module operating at 450 nm, yielding a functional and compact antimicrobial device. The optical fiber can be activated with a single click, triggering controlled NO release for at least 24 h. This light-induced NO delivery achieved over 90% reduction in bacterial viability for *S. aureus*, *S. epidermidis*, and *Proteus mirabilis*, without causing cytotoxic effects on mammalian cells.

#### 4.5.4. Ozone

Ozone is a powerful oxidizing agent that can damage the cell membranes of both Gram-positive and Gram-negative bacteria, leading to cell death. It is commonly used in dental clinics, both as a pre-treatment and post-treatment agent. As a pre-treatment, it prevents bacterial adhesion and effectively kills planktonic bacteria [197]. As a post-treatment, it can penetrate and disrupt the biofilm EPS matrix. This disruption not only leads to the breakdown of the biofilm structure but also increases bacterial susceptibility to antibiotic treatment or mechanical removal [198]. Coating biomaterials with ozone can modify surface properties, such as surface energy and hydrophobicity, making them less conducive to bacterial adhesion. In addition to dental clinics, ozone therapy is also employed in various other fields, including food processing and water treatment.

#### 4.5.5. Halogens

Halogens, such as fluorine, iodine, and chlorine, are well known for their antimicrobial properties. They disrupt bacterial cell membranes, inactivate essential enzymes, and interfere with DNA replication, thereby preventing or inhibiting bacterial growth and biofilm formation. For example, Tang et al. [199] designed a fluorinated alkoxyphosphazene surface that inhibited the adhesion of both Gram-positive and Gram-negative bacteria by 50- to 200-fold compared to untreated surfaces. Similarly, Inoue et al. [200] demonstrated that iodine-coated implants exhibit broad-spectrum antibacterial and antifungal activity. These implants were partially incorporated into the guidelines of the 2018 International Consensus Meeting on Musculoskeletal Infection due to their potential to prevent infections [201]. In addition to preventing bacterial adhesion, surfaces can be engineered to release halogens in a controlled manner. This approach enables a sustained release of the antimicrobial agent, providing long-lasting protection against biofilm formation. Wu et al. [202] developed an N-halamine coating that incorporates chlorine into a polymer matrix, which can be further applied to titanium scaffolds. The authors demonstrated that the coating not only releases chlorine in a controlled manner, providing long-lasting antibacterial efficacy, but can also self-regenerate itself after use.

### 4.6. Engineered Nanoparticles

#### Strategies to Penetrate the Biofilm

Implementing biomaterials that prevent bacterial adhesion and growth is the most effective way to combat biofilm-related infections. Biofilms can protect bacteria inside from chemical and physical attacks, making them 10- to 1000-times more resistant than their free-floating counterparts. As mentioned earlier, various methods have been created to design biomaterials that stop bacterial biofilm formation. However, these methods mostly focus on prevention, and if they fail, uncontrolled bacterial growth may occur, often requiring implant removal and additional surgery. These events can greatly reduce patient quality of life and significantly raise healthcare costs.

In 2014, Komnatnyy et al. [203] reported the development of a smart material capable of releasing an antibacterial compound only upon biofilm formation. Specifically, they engineered a coating in which ciprofloxacin is covalently bound to the surface via ester bonds sensitive to bacterial lipase hydrolases. These enzymes, particularly abundant in mature biofilms, act as biological triggers for drug release. Notably, no antibiotic was released in the absence of bacteria or a stable biofilm.

However, the success of antibiotic or particle penetration into biofilms depends on multiple factors, including particle size, shape, surface chemistry, and the physicochemical properties of the biofilm itself. Studies have shown that small particles (5–130 nm) penetrate biofilms more effectively than larger ones [204,205]. Additionally, spherical particles exhibit higher penetration rates compared to elongated forms [206]. Surface properties, such as hydrophobicity and electrostatic charge, also play a crucial role. Li et al. [207] demonstrated that neutral or anionic quantum dots (QDs) cannot penetrate *E. coli* biofilms, whereas cationic QDs can do so without obstruction. Interestingly, the diffusion behavior of cationic QDs also varies: hydrophilic QDs tend to cluster, while hydrophobic QDs are more evenly distributed within the biofilm, making them more suitable for therapeutic applications.

Over the past decade, bacteriophages, viruses that infect and kill bacteria, have emerged as promising agents for biofilm disruption. They offer several advantages over traditional antibiotics, particularly due to their high specificity and efficacy against antibiotic-resistant strains. Unlike antibiotics, bacteriophages do not harm human cells or disrupt beneficial microbiota, reducing the risk of collateral damage. The antibacterial and antibiofilm efficacy of bacteriophages has been extensively studied. In a rat model of prosthetic joint infection, Morris et al. [208] observed a five-fold reduction in *S. aureus* when using phages alone and a 22.5-fold reduction when phages were combined with vancomycin. Moreover, Yazdi et al. [209] reported a 5-log reduction in *P. mirabilis* biofilm on urinary catheters within three hours of phage treatment. When phages were combined with ampicillin, they achieved a 5-log reduction in just one hour and an 85% reduction in biofilm mass.

Mayorga-Ramos et al. [210], in their mini-review, highlighted the potential of encapsulating bacteriophages in liposomes, metallic nanoparticles (e.g., silver, gold, magnesium), and polymeric nanoparticles (e.g., poly(lactic-co-glycolic acid), PLGA). These delivery systems enhance phage stability, improve biofilm penetration, and enable targeted therapeutic delivery.

Despite their considerable therapeutic potential, several critical issues remain to be addressed regarding the use of bacteriophages. As observed with antibiotics, bacteria can develop resistance to bacteriophages. Known resistance mechanisms include mutations in phage receptor proteins, activation of restriction–modification systems, abortive infection strategies—where bacterial cells self-destruct to prevent phage propagation—and the acquisition of CRISPR-Cas-mediated immunity [211]. Such mechanisms may significantly limit the long-term efficacy of phage-based therapies.

An additional concern involves the role of lysogenic phages. As noted by Anomaly [212], lysogenic phages that integrate into bacterial genomes can promote antibiotic resistance in their bacterial hosts. This interaction may lead to a mutualistic relationship between bacteria and phages, potentially enhancing bacterial pathogenicity in humans.

Moreover, the immunogenicity of bacteriophages represents another important limitation. Podlacha et al. [213] reported that bacteriophages, due to their nucleoproteinaceous composition, are readily recognized by the host immune system. They may also modulate the adaptive immune response and induce the production of anti-phage antibodies [214], potentially affecting the efficacy and safety of repeated or systemic phage administration.

Lastly, phage therapy faces several regulatory hurdles due to its unique nature as a biological agent. A major limitation is the scarcity of clinical trials. According to ClinicalTrials.gov, only 37 clinical trials involving phage therapy (search term: bacteriophages) have been registered, of which just 2 have reached Phase 3 and only 1 has progressed to Phase 4 [215]. Other key concerns include issues related to intellectual property, the establishment of quality and safety standards, and the intrinsic variability in bacteriophage preparations [216].

Table 2 provides a summary of the strengths, weaknesses, mitigation strategies, and toxicity profiles of the methods discussed.

## 5. Future Perspective

This review focuses on the eradication of biofilms from biomaterial surfaces used in medical devices, emphasizing surface modification or functionalization with non-antibiotic agents to address the growing threat of multidrug-resistant bacterial pathogens. It provides comprehensive information on a wide range of EPS disruption strategies that have been investigated to prevent bacterial adhesion and remove established biofilm layers. Future studies should concentrate on the rational design of combinatorial therapies that employ synergistic, multi-target approaches. As most current investigations are limited to in vitro settings, future work can prioritize translational and preclinical in vivo models to assess long-term antifouling and biofilm eradication performance, host tissue compatibility, and immune responses. A deeper understanding of the molecular mechanisms by which these agents disrupt biofilms, along with their potential to influence bacterial resistance development, is essential. OMICs-based approaches (e.g., transcriptomics, proteomics) could be instrumental in elucidating these mechanisms. Furthermore, the absence of standardized protocols to evaluate non-antibiotic antibiofilm treatments limits reproducibility and clinical translation, meaning future efforts, therefore, will aim to develop standardized testing frameworks and clarify regulatory considerations for the application of these agents in medical device coatings and functionalized biomaterials.

## Figures and Tables

**Table 1:** Summary of other ESP-degrading enzymes with biofilm-dispersing activity.

Enzyme Classes	Enzyme Name	Component Target	Bacterial Target	Ref.
**Glycoside hydrolase**	Dextranase	α-1,6-glucan (dextran)	*Streptococcus mutans* (oral biofilm), *Lactobacillus* spp.,	[120,121,122]
	Mutanase	α-1,3-glucan (mutan)	*Streptococcus mutans*	[121]
	Cellulase	β-1,4-glucan (cellulose)	*Pseudomonas fluorescens*, *Salmonella enterica*, *E. coli*, *Komagataeibacter xylinus*	[123,124]
	Amylase	α-1,4-glucan (amylose)	General polysaccharide degradation (minor components)	[125]
	α- or β-Mannosidase	α- or β-mannose-containing polysaccharides	*Klebsiella* sp., *Pseudomonas* sp. (minor components)	[126,127]
**Proteases**	Subtilisin A (serine protease)	Biofilm structural proteins	*P. aeruginosa*, *S. aureus*	[128]
	Papain (cysteine protease)	Surface adhesin, extracellular protein	*S. aureus*, *S. epidermidis*	[129,130]
	Ficin (cysteine protease)	Biofilm structural and adhesive proteins	*S. aureus*, *S. epidermidis*, *S. mutans*	[131,132]
	Bromelain (cysteine protease)	Biofilm structural, adhesins	*S. aureus*, *S. epidermidis*, *E. coli*	[129,133]
	Metalloproteinases (zinc-dependent endopeptidase)	Fibronectin-binding proteins (FnBPs), biofilm-associated protein (Bap), accumulation-associated protein (Aap)	*S. aureus*, *S. epidermidis*, *Enterococcus faecalis*	[133,134]
	Alkaline protease (serine protease)	FnBPs, Bap, Aap	*S. aureus*, *S. epidermidis*, *E. coli*	[135]
**Deoxyribonucleases**	Streptodornase (DNase B)	Extracellular DNA (eDNA)	Gram-positive and Gram-negative bacteria	[136,137]
	NucB	eDNA	*E. coli*, *Bacillus subtilis*, *Micrococcus luteus*	[76,138]
	Micrococcal nuclease	Calcium-dependent endo-exonuclease	*S. aureus*, *S. epidermidis*, *P. aeruginosa*, *B. subtilis*	[139]
	Serratia nuclease (NucA)	Single- and double-stranded eDNA	*E. coli*, *Bacillus subtilis*	[138]

**Table 2:** Antibacterial strategies: strengths, weaknesses, mitigation strategies, and toxicology profile.

	Strengths	Weaknesses	Mitigation Strategies	Toxicity Profile
**Biofilm dispersing enzymes**	High specificity Less likely to promote resistance development Enhanced antimicrobial efficacy	Enzyme instability and poor retention Limited efficacy against mature biofilms Delivery challenges High cost and limited scalability Narrow spectrum of activity	Enzyme encapsulation in protective carriers can enhance stability, protect against degradation, and improve delivery to infection sites [217] Enzyme engineering to improve stability and activity [218] Combining different enzymes or co-administering with antibiotics can increase effectiveness [219]	Glycoside hydrolases (GHs) may be toxic to human cells in vitro [217] DNases can cause DNA fragmentation in eukaryotic cells [220] Proteases may damage eukaryotic cell membranes and induce apoptosis [221]
**Chelating agents**	Reduced resistance development compared to other methods Inhibition of bacterial growth Biofilm disruption Potential synergistic effects High versatility	Low specificity (cannot distinguish between prokaryotic and eukaryotic cells) Efficacy depends on bacterial species and their metal requirements Delivery challenges	Combination with antibiotics to overcome resistance [222] Development of agents targeting only prokaryotic cells [223] Nanoparticle-based delivery to improve targeting [224]	May deplete essential metals in host cells, leading to dysfunction and toxicity [225] Potential interference with host metal-dependent enzymes, causing oxidative stress [226]
**Quorum sensing inhibitors**	Reduction of antibiotic resistance Targeting of bacterial virulence factors Availability of natural product-based options	Limited specificity Susceptibility to washout Challenges in clinical translation Limited efficacy	Developing QSIs with high specificity can minimize off-target effects and improve efficacy [227] Nanoparticle-based systems can enhance delivery and retention [228] Further research into pathogen-specific QS pathways to design targeted inhibitors [229]	Potential interference with similar signaling pathways in human cells [230]
**Biosurfactants**	Biodegradable Versatile Stable under extreme conditions Modifiable via genetic engineering	Low production yield High production costs Limited understanding and research Lack of production and safety standards	Genetic engineering and nanotechnology approaches to improve production and properties [231] Use of agricultural waste to reduce production costs [232]	Toxicity depends on type, concentration, exposure, and environmental conditions
**Oxidizing agents**	Broad-spectrum antimicrobial activity Low likelihood of resistance development Rapid disinfection High versatility	Formation of harmful by-products Short half-life of some agents Environmental concerns	Combination with other antimicrobials to improve efficacy and reduce resistance risk [233] Encapsulation in delivery systems for controlled release	Induction of oxidative stress Mitochondrial dysfunction Accumulation in tissues or organs [234] Potential to trigger inflammation

## Data Availability

The dataset is available on request from the authors.

## Abbreviations

The following abbreviations are used in this manuscript:

WT	Wild type
EPS	Extracellular polymeric substances
C-DI-GMP	Cyclic di-guanosine monophosphate
QS	Quorum sensing
AHL	N-acylated homoserine lactone
AI	Autoinducers
SpA	S. Aureus protein a
PVL	Panton-Valentine leucocidin
CVC	Central venous catheter
CAUTI-	Catheter-associated urinary tract infections
VAP	Ventilator-associated pneumonia
ICU	Intensive care units
MRSA	Methicillin-resistant S. Aureus
PGA	Poly-N-acetyl-glucosamine (PGA)
CA	Colanic acid
dPNAG	De-N-acetylated poly β-(1,6)-N-acetyl-d-glucosamine
CF	Cystic fibrosis
OMV	Outer membrane vescicles
DspB	Dispersin B
PNAG	Poly-(1→6)-β-N-acetylglucosamine
Aap	Accumulation-associated protein
EbpS	Elastin-binding protein
eDNA	Extracellular DNA
PMNs	Polymorphonuclear leukocytes
eRNA	Extracellular RNA
NTHI	Non-typeable Haemophilus influenzae
LPS	Lipopolysaccaride
ALU	Aluminium
SR	Silicon rubber
PET	Polyethylene terephthalate
DNases	Deoxyribonucleases
GH20	Glycoside hydrolase family 20
PGA	Poly-N-acetyl glucosamine
MagR	Magnetoreceptor
LBL	Layer by layer
PAH	Poly(allylamine hydrochloride)
PMAA	Poly(methacrylic acid)
BC	Bacteria cellulose
PCD	Phenylboronic acid-modified carbon dots
ROS	Reactive oxygen species
RhDNAse I	Recombinant human dnase I
Ti	Titanium
AC-EPD	Alternating current electrophoretic deposition
FDA-	Food and Drug Administration
EDTA	Ethylenediamine-tetra-acetic acid
TSC	Trisodium citrate
EGTA	Ethylene glycol-bis (β-aminoethyl ether)-N,N,N’,N’-tetra-acetic acid
DFO	Desferrioxamine
DFP	Deferiprone
AHL	N-acyl homoserine lactones
PVD	Pyoverdine
PDMS	Polydimethylsiloxane
SLA	Silicone discs with lactonic sophrolipids
RL	Rhamnolipid
NO	Nitric oxide
AuNPs-	Gold nanoparticles
Ga	Gallium
AgNPs	Silver nanoparticles
AgNP–PNCs	Agnp–polymer nanocomposites
EDS	Dispersive X-ray Spectroscopy
Z	Zirconia
ZGa	Gallium-doped zirconia
GO	Graphene oxide
SNAP	S-nitroso-N-acetylpenicillamine
QD	Quantum dot
PLGA	Poly(lactic-co-glycolic acid)
